# Nanoconfinement induced crystal orientation and large piezoelectric coefficient in vertically aligned P(VDF-TrFE) nanotube array

**DOI:** 10.1038/srep09790

**Published:** 2015-05-12

**Authors:** Weng Heng Liew, Meysam Sharifzadeh Mirshekarloo, Shuting Chen, Kui Yao, Francis Eng Hock Tay

**Affiliations:** 1 Institute of Materials Research and Engineering (IMRE), A* STAR (Agency for Science, Technology and Research) 3 Research Link, 117602 (Singapore); 2 Department of Mechanical Engineering National University of Singapore Kent Ridge, 119260 (Singapore)

## Abstract

Vertically aligned piezoelectric P(VDF-TrFE) nanotube array comprising nanotubes embedded in anodized alumina membrane matrix without entanglement has been fabricated. It is found that the crystallographic polar axes of the P(VDF-TrFE) nanotubes are oriented along the nanotubes long axes. Such a desired crystal orientation is due to the kinetic selection mechanism for lamellae growth confined in the nanopores. The preferred crystal orientation in nanotubes leads to huge piezoelectric coefficients of the P(VDF-TrFE). The piezoelectric strain and voltage coefficients of P(VDF-TrFE) nanotube array are observed to be 1.97 and 3.40 times of those for conventional spin coated film. Such a significant performance enhancement is attributed to the well-controlled polarization orientation, the elimination of the substrate constraint, and the low dielectric constant of the nanotube array. The P(VDF-TrFE) nanotube array exhibiting the unique structure and outstanding piezoelectric performance is promising for wide applications, including various electrical devices and electromechanical sensors and transducers.

Poly(vinylidene fluoride-co-trifluoroethylene) (P(VDF-TrFE)) is currently the most technically important piezoelectric polymer with large piezoelectric coefficient and low dielectric loss.^1^,^2^ As compared to piezoelectric ceramics, piezoelectric polymers have the advantages of high flexibility and low acoustic impedance, making them competitive for ultrasound sensing in organic mediums^3^ and energy harvesting from mechanical motions in natures^4^ and human movements.^5^ Thin films of P(VDF-TrFE) have been extensively studied in the past decades^6^ and found various applications, including tactile sensors^7^ and ultrasonic transducers.^8^ Apart from the thin film structure, various P(VDF-TrFE) nanostructures have also been demonstrated, including nanocell,^9^ nanorod^10^ and nanotube.^11^ These nanostructures have attracted much research interest recently due to the potential ability to control the structures and properties of piezoelectric polymers at nanoscales.

Piezoelectric one dimensional (1D) nanostructures have been considered as potential candidates for applications such as sensing,^12^ photonics,^13^ piezotronics^14^ and energy harvesting.^15^,^16^ A notable example is the illustration of zinc oxide nanowires energy harvesters by utilizing the coupled piezoelectric and semiconducting effects of zinc oxide nanowires.^17^ Many other one-dimensional nanostructures, including nanotubes (NT), nanofibers (NF) and nanorods (NR), have also been demonstrated with properties different from their bulk counterparts.^18^,^19^ Several studies have reported anisotropic behaviors of crystal orientations in geometrically constrained 1D polymeric nanostructures.^20^,^21^,^22^ Given the strong dependency of piezoelectricity on orientation of crystal structures,^23^ appropriately controlled crystal orientation in 1D nanostructure is a potentially effective strategy to enhance the piezoelectric performance. Hence, study on P(VDF-TrFE) nanotube array is of great importance and interest in both fundamental and application aspects.

1D polymer nanoarrays are often fabricated by nanoimprinting^9^,^24^ and template-assisted infiltration.^11^,^25^ Template-assisted method is based on the wetting capability of polymer melts or polymer solution on the wall of nanoporous template due to the high-surface energy of template walls.^26^ Anodized alumina membrane (AAM) is a commonly used template due to the controllable pore size and depth, relative high surface free energy and ease of preparation. Although the fabrication of P(VDF-TrFE) 1D nanostructures have been reported recently,^10^,^11^,^27^ the crystal structure and control of crystal and polarization orientation are not in-depth studied or realized, and thus the potential of the piezoelectric polymer 1D nanostructures is not convincingly demonstrated. Among the various nanostructures, the fabrication of vertically aligned 1D polymer nanotubes with high aspect ratio is challenging due to the low stiffness of the hollow polymers. Entanglement and formation of nanotube’s bundles always occur in the previous efforts when the AAM template is removed. In this work, we have successfully produced vertically aligned P(VDF-TrFE) nanotube array with the desired crystal and polarization orientation using the AAM template. We controlled the etching process of AAM dedicatedly to produce an alumina matrix which provides mechanical support to the straight-standing P(VDF-TrFE) nanotubes without entangling and without sacrificing piezoelectric properties. The obtained P(VDF-TrFE) nanotube array exhibited piezoelectric coefficient significantly higher than that of monolithic film on a substrate. We further demonstrated the correlation between the crystal orientation and the significantly enhanced piezoelectric performance observed in our P(VDF-TrFE) nanotube array.

## Results and Discussions

### Morphology

**Figure 1:**
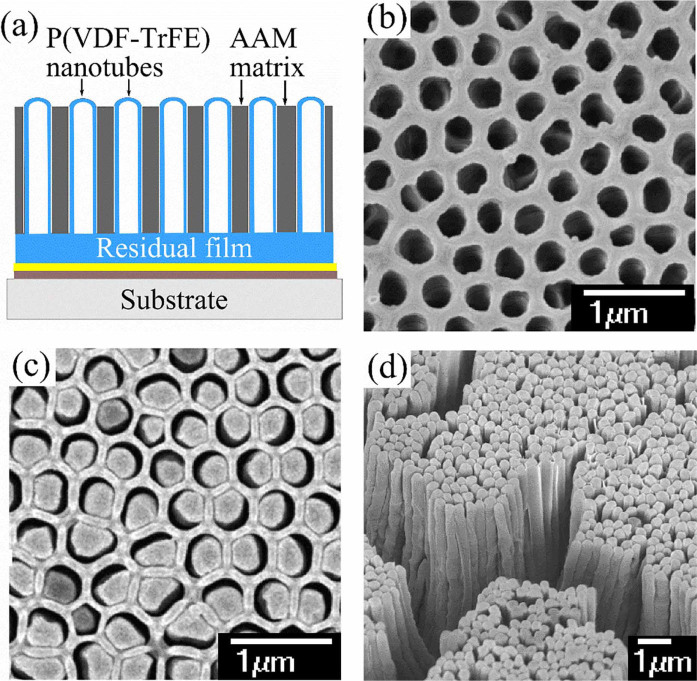
(a) Schematic drawing of the nanotube array embedded in AAM matrix adhered on glass substrate. (b) AAM template coated with P(VDF-TrFE) after removing residual film by RIE (bottom view). (c) Exposed P(VDF-TrFE) nanotube array embedded in AAM matrix (top view). (d) Formation of P(VDF-TrFE) nanotube’s bundles when AAM is removed.

The overall procedure of fabricating P(DVF-TrFE) nanotube array is shown in Supplementary Scheme S1. The schematic diagram of the P(VDF-TrFE) nanotube array is shown in Fig. 1a. The hollow structure as observed in Fig. 1b shows the open end of the nanotubes formed in AAM template after deposition of 15 cycles and removal of residual film using RIE. Figure 1c shows the morphology of P(VDF-TrFE) nanotube array embedded in AAM template observed with scanning electron microscope (SEM). The wall thickness of P(VDF-TrFE) nanotubes reached about 60 nm after 15 cycles deposition.

Figure 1c presents the close end of the exposed nanotube array (top view) in the remaining AAM matrix. The polymer melt spontaneously wets the inner walls of the alumina nanopores and forms nanotube structure due to the lower surface tension of P(VDF-TrFE) melt at 250 °C.^11^ The remaining AAM matrix supports the P(VDF-TrFE) nanotubes to promote vertically standing nanotubes and prevents formation of nanotubes bundle. The lack of AAM matrix causes the nanotubes to form bundle as shown in Fig. 1d due to the low stiffness of the polymers. The interstice between P(VDF-TrFE) nanotubes and AAM walls suggests that the nanotubes are not clamped to the AAM walls. Such configuration is crucial to achieving superior piezoelectric performance, otherwise the axial movement of nanotubes along the AAM nanopores will be restricted.

### Crystal Structure

**Figure 2:**
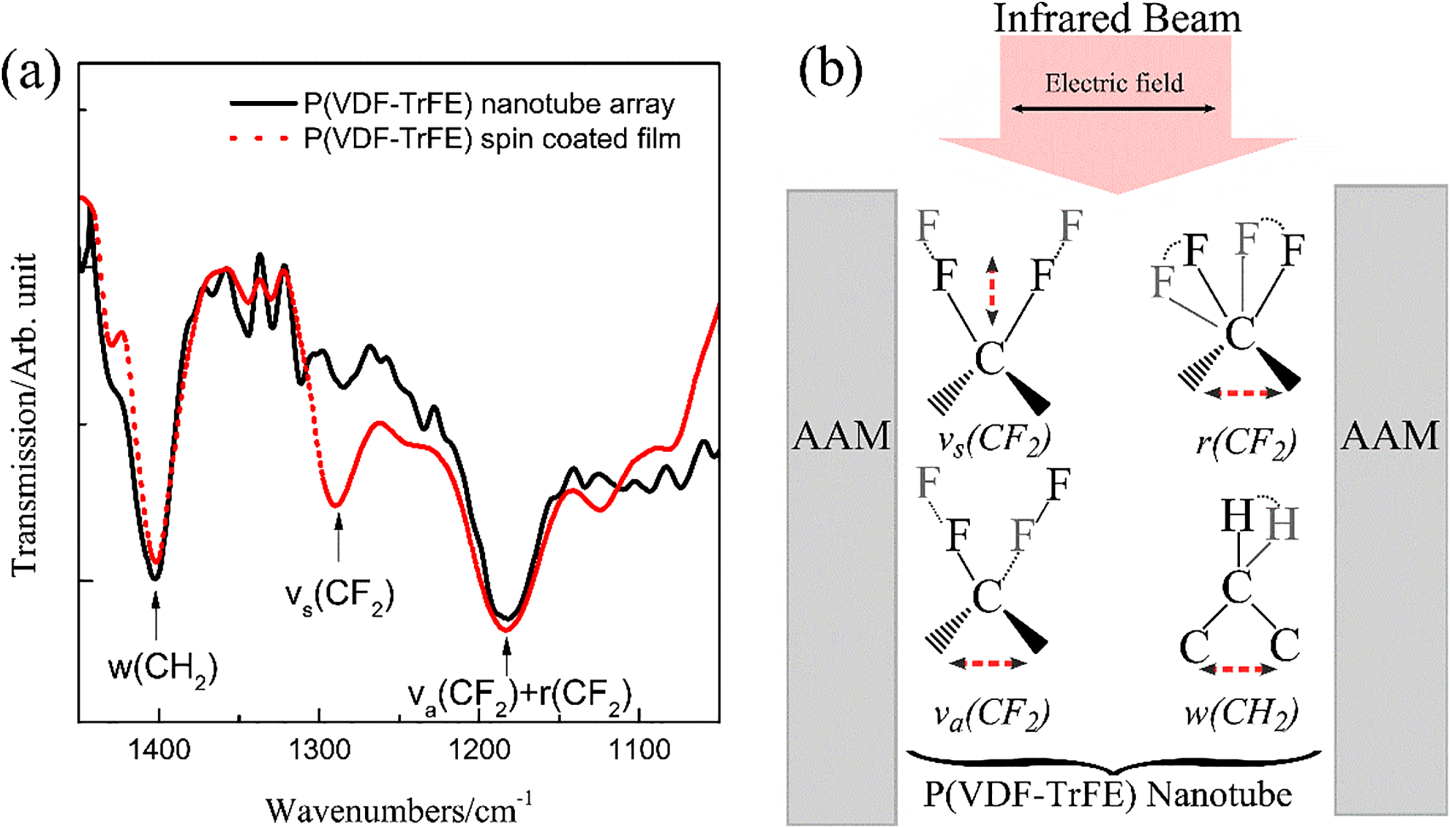
(a) FTIR spectra of the nanotube array and spin coated film (normalized to absorption bands at 1400 cm^−1^). b) Orientation of infrared beam and different vibrational modes. Red dashed lines besides the vibrational modes indicate the orientation of activation electric field for respective vibrational modes.

The XRD spectrum of P(VDF-TrFE) nanotube array (see Supplementary Figure S1) showed a prominent peak at 19.8° (corresponding to (110) and (200) Bragg reflections of the β phase)^28^ . This indicates that the P(VDF-TrFE) nanotube array is dominantly ferroelectric phase. Fourier-transform infrared reflection microscopy (FTIR) was used to further examine the crystal structure of P(VDF-TrFE) nanotubes. The infrared absorbance is highly dependent on the relative orientation of electric field in infrared and the absorbing molecules. The infrared absorbance is zero when the electric field vector of infrared beam and the dipole transition moments of the materials are oriented perpendicularly. The electric field of the infrared beam in our FTIR experiment is in plane with the sample’s surface (infrared beam is perpendicular to the sample surface plane). As shown in Fig. 2a, the absorption band at 1288 cm^−1^ (symmetric stretching vibration of CF_2_ with dipole transition moment parallel to the polar *b* axis, *v_s _(CF_2_)*) is virtually extinct in the nanotube array sample. This phenomenon indicates that the polar *b* axis of most P(VDF-TrFE) crystals are oriented perpendicular to the sample surface. On the contrary, the absorption bands at 1400 cm^−1^ (wagging vibration of CH_2 _with dipole transition moments parallel to the chain *c* axis, *w (CH_2_)*) and 1187 cm^−1^ (antisymmetric stretching, *v_a _(CF_2_)* and rocking vibration, *v_a_(CF_2_)* of CF_2_ with dipole transition moments parallel to the *a* axis) appear strongly in the nanotube array sample, suggesting that the *a* and *c* axis of most polymer crystals are oriented perpendicular to the nanotube long axis.

**Figure 3:**
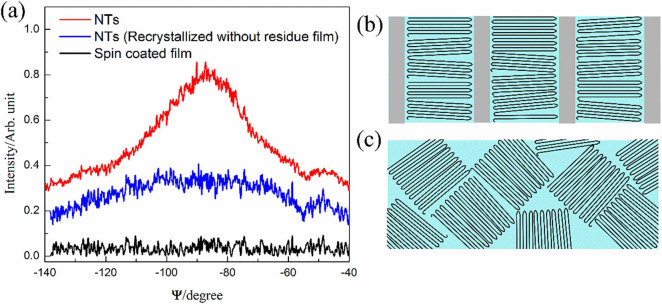
(a) The Ψ dependence of the XRD peak for the planes (200) and (110) of the nanotubes (NTs), nanotubes after recrystallization without residual film, and spin coated film. Schematic drawing of (b) preferentially oriented polymer chains in nanotubes and (c) randomly oriented polymer chains in spin coated film.

To further verify this finding, we investigated the Ψ dependence of the XRD peak intensity for the crystal planes (200) and (110) of the nanotube array sample as compared to spin coated P(VDF-TrFE) film. Ψ is the incline angle between the normal of the crystal plane and the sample surface plane. As shown in Fig. 3a, (200) and (110) diffraction peak of the nanotube array exhibits much stronger Ψ dependence than the spin coated film, showing the maximum at around Ψ = −90°. Since the polymer chains lie in the crystal planes of (200) and (110) of β phase P(VDF-TrFE), the Ψ dependence of nanotube array further shows that the polymer chains are perpendicular to the nanotube long axis, which is in agreement with the FTIR results.

As suggested by previous study on crystallization of PVDF homopolymer in nanopores,^29^ the preferred orientation of polymer chains in P(VDF-TrFE) nanotubes can be attributed to the initial random nucleation of P(VDF-TrFE) crystals in the residual film followed by selective directional polymer growth due to the confinement effect imposed by the nanopores. Crystallization of polymer melt generally occurs through heterogeneous nucleation since homogeneous nucleation requires much larger supercooling to overcome the intrinsic energy barrier for nucleation. Heterogeneous nucleation predominantly occurs at impurities (foreign particles) due to the much lower energy barrier. The typical density of impurities in P(VDF-TrFE) is estimated to be in the order of 10^−5^ μm^−3^.^30^ The volume of P(VDF-TrFE) in the nanotube with 350 nm in diameter, 60 nm wall thickness and 4 μm long is only 0.22 μm^3^. The average number of impurities in a nanopore is calculated to be 2.2 × 10^−6^, which indicates that impurity particles are rarely present in the nanopore. Although the density of foreign particles in the P(VDF-TrFE) may vary due to synthesis conditions, the average number of impurities is still several orders smaller than one in a nanopore. Due to the limited number of impurities in the polymer melt inside the AAM nanopores, heterogeneous nucleation is believed to be ineffective and suppressed inside the AAM nanopores.

Hence, heterogeneous nucleation should predominantly occur in the residual film and proceed by growth of lamellae, and this statement is supported by our DSC results (see Supplementary Figure S2). Polymer lamellae in the spherulites in residual film grow in all directions with the fastest crystallographic growth direction (which is along the *<hk0>* direction for P(VDF-TrFE)^31^) points radially outward. The growth of lamellas in the residual film does not have any substantially preferred orientation until they hit the nanopores of AAM. Lamellae with fastest growth direction not parallel to the nanopore long axes are suppressed since the growth of lamellae in such directions is hindered by the nanopore walls. Thus, a kinetic selection mechanism in the residual film followed by unidirectional growth along the nanopores results in macroscopic nanotube array with preferred crystal orientation with polymer chains aligned perpendicular to the nanopore axis. To further verify this mechanism, the P(VDF-TrFE) nanotube array without the residual film is heated up to 250 °C for recrystallization. As shown in Fig. 3a, P(VDF-TrFE) nanotube array recrystallized without residual film exhibits a much weaker Ψ dependence, which indicates the random orientation of polymer chain in the nanotubes. This suggests that crystallization starting from the residual film is essential to obtain the desired polarization alignment along the longitudinal axis of the obtained P(VDF-TrFE) nanotubes.

### Ferroelectric Properties

**Figure 4:**
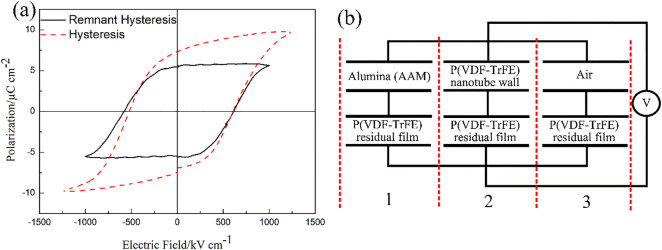
(a) Polarization – electric field (P-E) hysteresis loop of P(VDF-TrFE) nanotube array. (b) Schematic of electrical model to calculate the total polarization.

The ferroelectric characterization was performed on the P(VDF-TrFE) nanotube array with residual film which provides a uniform surface for bottom electrode. The voltage was applied on the top-bottom electrodes for creating an electric field across the length of the nanotubes. Figure 4 shows the polarization-electric field (P-E) hysteresis of the P(VDF-TrFE) nanotube array. Remnant hysteresis was obtained after subtracting the hysteresis with the non-remnant component, which is mostly due to leakage current.^32^ The apparently observed remnant polarization *P_r_*, of 5.10 µC cm^−2^ is lower than the previously reported value of 8.35 µC cm^−2^ 33 for P(VDF-TrFE) thin film. The lower remnant polarization can be explained by the hollow structure of the nanotubes and presence of alumina in the P(VDF-TrFE) nanotube array.

In order to analyze the ferroelectric polarization of the nanotube array, the total polarization is modeled using an electric circuit as shown in Fig. 4b**.** The effective remnant polarization is the sum of the polarization of three regions. Due to the presence of alumina and air in series connection with P(VDF-TrFE), the low charge carrier density of these materials causes insufficient compensation charges to move into close proximity of ferroelectric surfaces to completely eliminate the depolarization field.^34^ Hence, the incomplete shielding of depolarization field destabilizes and reduces the ferroelectric polarization in the nanotube array. The calculated effective remnant polarization based on this model without considering the preferred crystal orientation is 3.35 µC cm^−2^ (calculation details in supplementary information) which is significantly lower than the experimentally observed value.

**Figure 5:**
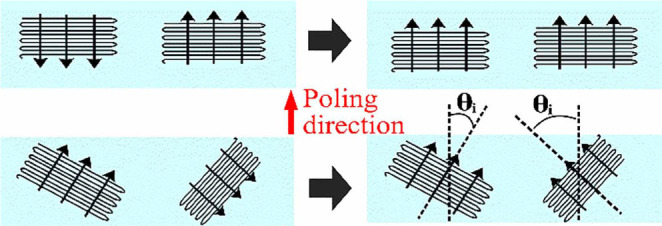
Rotation of dipoles in P(VDF-TrFE) in response to the applied electric field. The maximum effective polarization in poling direction is a function of *θ_i_*, the angle between poling direction and normal of polymer chains. The smaller the angle *θ_i_*of the polymer chains, the higher the contribution to the observed polarization.

It is known that the polarization in piezoelectric polymer can be enhanced by the preferred crystal orientation.^35^ Dipoles in vinylidene fluoride polymers can only rotate around the polymer chain (c-axis) in response to the applied electric field during the poling process as shown in Fig. 5. Hence, the effective remnant polarization is a function of angular distribution of polymer crystal relative to the applied electric field.

Our previous quantitative calculation have showed that the polarization in the P(VDF-TrFE) film with randomly oriented polymer chains is two-third of that in the film with all polymer chains aligned perpendicular to the applied electric field.^35^ Hence, the remnant polarization in the P(VDF-TrFE) nanotubes is enhanced by approximately a factor of 1.5 due to preferred orientation of polymer chains and estimated to be approaching 11 µC cm^−2^. The calculated effective remnant polarization for P(VDF-TrFE) nanotube array with residual film considering the preferred crystal orientation effect is 4.35 µC cm^−2^, which is closer to the experimental value of 5.10 µC cm^−2^ and indicating the enhancement in polarization due to preferred crystal orientation in nanotubes. The even higher experimental value can be due to the excessive infiltration of P(VDF-TrFE) in the nanopores during the melt-wetting process.

### Piezoelectric Properties

**Figure 6:**
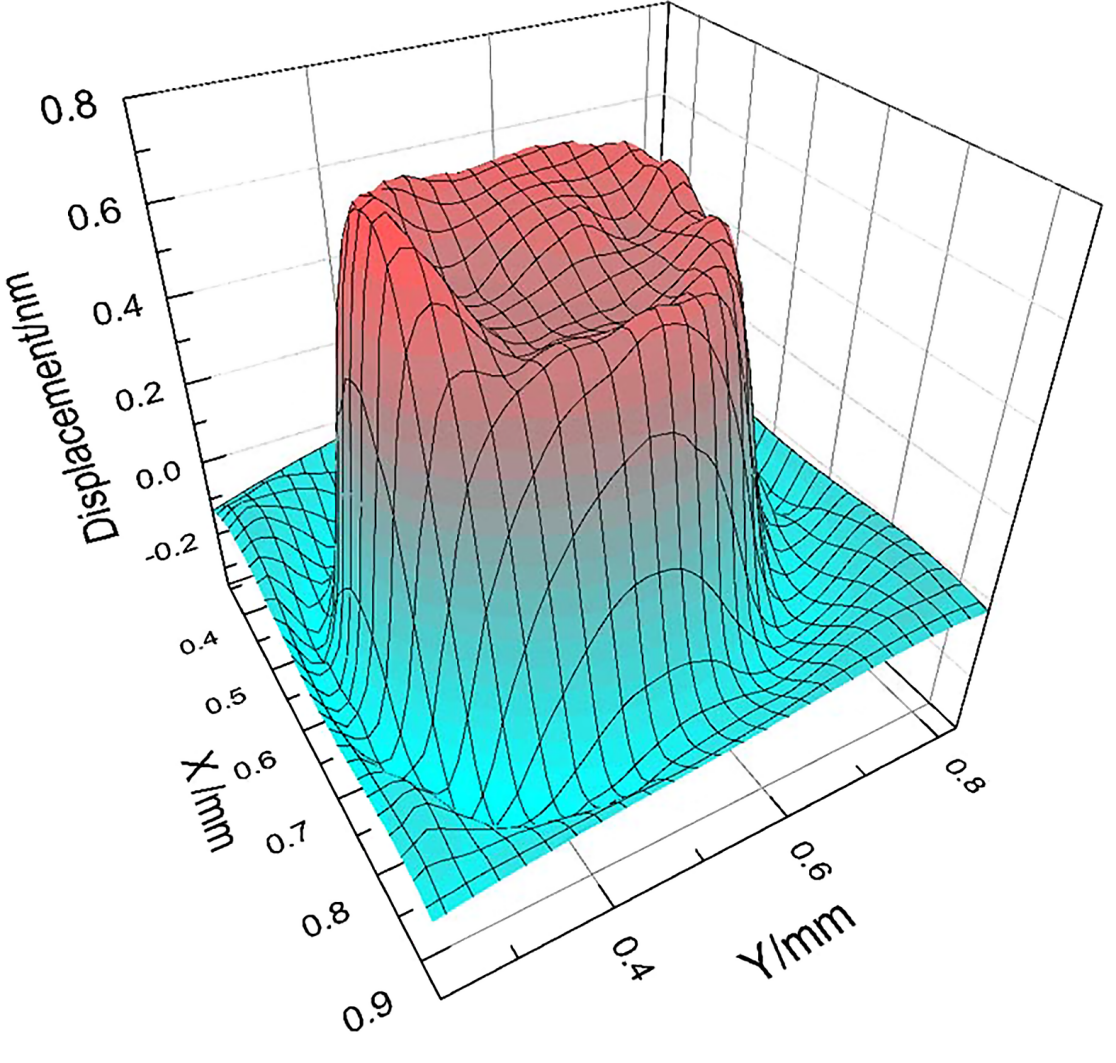
Three dimensional drawing of the instantaneous displacement data when the displacement magnitude reaches the maximum under the sine-wave driving electrical signal. The trough observed on the maximum displacement surface is due to the compression force exerted by the probe.

The effective piezoelectric coefficient *d_33_* of the nanotube array was measured with a laser scanning vibrometer (LSV) under a unipolar ac signal of amplitude 20 V at 3 kHz. LSV monitors the vibration of the whole surface (circle with diameter of 1 mm) instead of a single point on the samples to produce a reliable piezoelectric measurement.^36^ Figure 6 presents the three dimensional drawing of the instantaneous displacement data when the displacement magnitude reaches the maximum under the sine-wave driving electrical signal. The effective piezoelectric coefficient *d_33_*of the P(VDF-TrFE) nanotube array was recorded to be as high as −35 pm V^−1^, which is about 97% higher than the previous results on 1 µm-thick spin coated P(VDF-TrFE) film (−17.8 pm V^−1^ 33).

The effect of preferred crystal orientation on piezoelectricity of P(VDF-TrFE) can be analyzed based on the dimensional effect,^37^ which is the dominant mechanism for piezoelectricity in P(VDF-TrFE). The model assumes that the magnitude of the individual dipole moments in the P(VDF-TrFE) remains constant when external stress and electric field are applied. The piezoelectric response mainly originates from the macroscopic dimensional change of the materials instead of the stress-induced changes in dipole moment. The dimensional change causes change in density of dipole moments which induces polarization change and results in piezoelectric effect. Assuming the deformation only occurs in thickness mode, the piezoelectric constant based on the dimensional effect can be expressed as^38^


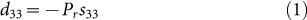
where *P_r_* is the remnant polarization and *s_33_* is the elastic compliance in the thickness direction. Based on this equation, the enhancement in piezoelectric constant due to the preferred crystal orientation can be calculated by considering the improved remnant polarization of P(VDF-TrFE) nanotubes. Given the value of *s_33_*for P(VDF-TrFE) is 0.4 × 10^−9^ m^2^ N^−1^ and *P_r_*is 11 µC cm^−2^ in nanotubes, the calculated piezoelectric constant for P(VDF-TrFE) nanotubes with preferred orientation is −44 pm V^−1^, which is 30% higher than the free standing P(VDF-TrFE) bulk film (−33.5 pm V^−1^ 39). Hence, the overall effective piezoelectric coefficient of the P(VDF-TrFE) nanotube array with residual film can be estimated using the model as shown in Fig. 4b. Due to the similarity in thickness of residual film and nanotube array, the effective piezoelectric constant is shown to be


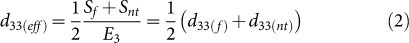
where *S_f_* and *S_nt_* are the piezoelectric strain, *d_33(f)_*and *d_33(nt)_* are the piezoelectric coefficient of the residual film and nanotube array respectively. The piezoelectric coefficient of the residual film, *d_33(f)_*, is estimated to be −17.8 pm V^−1^ due to the substrate clamping effect whereas the piezoelectric constant of the nanotube array, *d_33(nt)_*, is shown to be −44 pm V^−1^. Substrate clamping effect is assumed to be negligible for nanotube structure due to the high aspect ratio (length-to diameter ratio is about 11). The calculated effective piezoelectric coefficient for P(VDF-TrFE) nanotube array with the residual film is −31 pm V^−1^, which is close to the experimental value of −35 pm V^−1^. This shows that the piezoelectric constant of P(VDF-TrFE) nanotubes is enhanced by the preferred crystal and polarization orientation of P(VDF-TrFE).

The piezoelectric voltage coefficient g_33_ can be calculated by


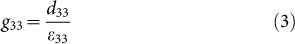
where *ε_33_* is the dielectric constant, which is 7.7 for P(VDF-TrFE) nanotube array as measured at 1 kHz, which is lower than the spin coated film of 13.2.^33^ The lower dielectric constant of P(VDF-TrFE) nanotube array is due to the existence of air and alumina in the nanotube array which have lower dielectric constants than the P(VDF-TrFE). The calculated *g_33_*value for P(VDF-TrFE) nanotube array sample is 513.4 mmV N^−1^, which is dramatically larger than various currently available piezoelectric materials as listed in Table 1. The *g_33_* value for P(VDF-TrFE) nanotube array sample is 26 times of lead zirconate titanate (PZT-5H), 18 times of potassium sodium niobate (KNN) and 3.8 to 3.4 times of spin-coated film of PVDF and P(VDF-TrFE). The huge piezoelectric voltage coefficient is desired for sensing application as sensitivity of the sensor is dependent on the voltage output in response to the applied stress.

## Conclusion

We have demonstrated the fabrication of vertically aligned piezoelectric P(VDF-TrFE) nanotube array comprising nanotubes with an outer diameter of ~350 nm and a wall thickness of ~60 nm embedded in anodized alumina membrane matrix without entanglement. The crystallographic polar axes of the P(VDF-TrFE) nanotubes are found to orient along the nanotubes long axes. Such a desired crystal orientation is due to the kinetic selection mechanism for lamellae growth confined in the nanopores. The preferred crystal orientation in nanotubes leads to huge piezoelectric coefficients of the P(VDF-TrFE). The obtained *d_33_* is −35 pm V^−1^, which was about 1.97 times of that for the spin coated film with solid substrate (−17.8 pm V^−1^), and g_33_ is about 3.40 times of spin coated film. Such a significant performance enhancement is attributed to the well-controlled polarization orientation, the elimination of the substrate constraint, and the low dielectric constant of the nanotube array. The P(VDF-TrFE) nanotube array exhibiting the unique structure and outstanding piezoelectric performance is promising for wide applications, including various electrical devices and electromechanical sensors and transducers.

## Methods

### Fabrication of P(VDF-TrFE) nanotube array in AAM template

The one-end sealed AAM template with pore size 300 – 350 nm and depth of 4 µm was fabricated using two-step anodization method. High purity Al foil (99.999%, Goodfellow) was anodized in 0.2 wt% H_3_PO_4 _(H_2_O : ethanol = 1:1 in volume) under a constant applied voltage of 180 V for 5 h at 4°C. The alumina layer formed at the surface of Al foil after first step of anodization was etched in 6 wt% H_3_PO_4_ and 1.8 wt% HCrO_4 _solution. Second anodization was performed to obtain a highly ordered AAM layer. The nanopores of AAM were enlarged by etching in 5 wt% H_3_PO_4 _solution at 50 °C for 25 min. The AAM template had an average wall thickness of 70 nm and pore size of 350 nm. A 10 wt% P(VDF-TrFE) (72/28, Solexis) solution was prepared by dissolving the P(VDF-TrFE) pellets in a mixed solvent of dimethyl-formamide (DMF) and acetone. The P(VDF-TrFE) solution was spin-coated on the AAM and dried at 100 °C. The sample was heated to 250 °C to melt the polymer. The polymer melt wetted the AAM template and infiltrated into the nanopores by capillary force. The deposition was repeated for 15 cycles to obtain the desired nanotube wall thickness. The final structure was consisting of P(VDF-TrFE) nanotube array attached to a residual P(VDF-TrFE) film. Bottom gold electrode was deposited on the residual film by e-beam evaporation and the sample was attached on a glass substrate using adhesive epoxy. Residual aluminum was removed with copper chloride solution (in hydrochloric acid). The nanotubes were exposed by controlled etching of the AAM in 5 wt% H_3_PO_4 _solution. Gold top electrodes were then deposited on the top of nanotube array with by e-beam evaporation.

### Characterization of P(VDF-TrFE) nanotube array

Morphology of the P(VDF-TrFE) nanotube array was investigated with field emission scanning electron microscopy (FESEM, JSM-6700F, JEOL). For XRD and FTIR measurements, the residual P(VDF-TrFE) film was removed by reactive-ion etching (RIE). The crystalline structure of P(VDF-TrFE) nanotube array without residual film was examined using X-ray diffraction (XRD) (D8-ADVANCE, Bruker AXS GmbH, Karlsruhe) and transmission mode Fourier Transform Infrared Spectroscopy (FTIR) (Spectrum 2000, Perkin-Elmer, Norwalk, CT). The polarization versus electric field (P-E) hysteresis loops were measured with a standard ferroelectric testing unit (Precision Premier II, Radiant Technology) connected to a high voltage interface. The effective piezoelectric constant *d_33_*was measured with a laser scanning vibrometer (OFV- 3001-SF6, PolyTech GmbH) after the samples were poled under 800V. The crystallization temperature was measured by differential scanning calorimetry (DSC) (DSC 1, Mettler Toledo).

## Supplementary Material

Supplementary Information Supplementary Information

## Figures and Tables

**Table 1:** Comparison of piezoelectric properties of various piezoelectric materials with P(VDF-TrFE) nanotube array

		PVDF^33^	P(VDF-TrFE)^33^	PZT-5H^40^	KNN^41^	P(VDF-TrFE) Nanotube array
*d_33_*	pm V^−1^	−15.0	−17.8	593	74	−35
*ε_r_*		12.5	13.2	3400	295	7.7
*g_33_*	mmV N^−1^	−135.5	−152.3	19.7	28.3	−513.4
