# Supplementary material for: Nanoconfinement induced crystal orientation and large piezoelectric coefficient in vertically aligned P(VDF-TrFE) nanotube array
Source: Sci Rep. 2015 May 12;5:09790. doi: 10.1038/srep09790 (PMC4434347; doi:10.1038/srep09790)
Supplement: Supplementary Information [file srep09790-s1.pdf]

Supplementary Information

Nanoconfinement Induced Crystal Orientation And Large Piezoelectric Coefficient In Vertically Aligned P(VDF-TrFE) Nanotube Array

Weng Heng Liew, Meysam Sharifzadeh Mirshekarloo, Shuting Chen, Kui Yao, and Francis Eng Hock Tay*

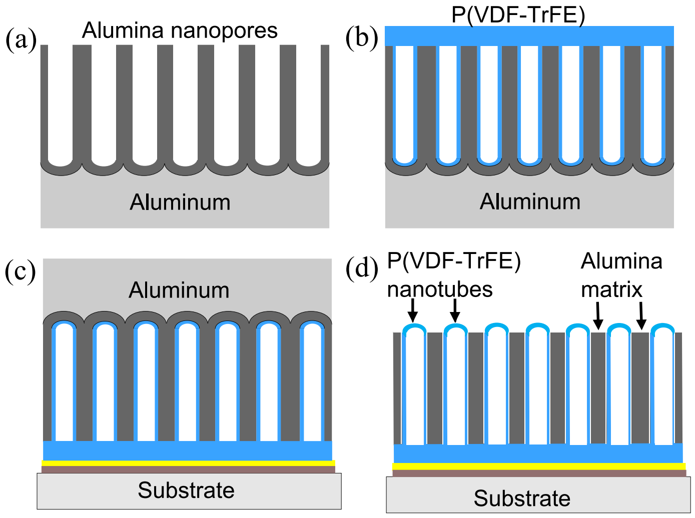

Scheme S1. Schematic illustration of a) AAM template; b) AAM template with spin coated P(VDF-TrFE) nanotube array; c) deposition of bottom Au electrode and attachment on glass substrate; and d) dissolution of aluminum and controlled etching of AAM template to expose the nanotube array.

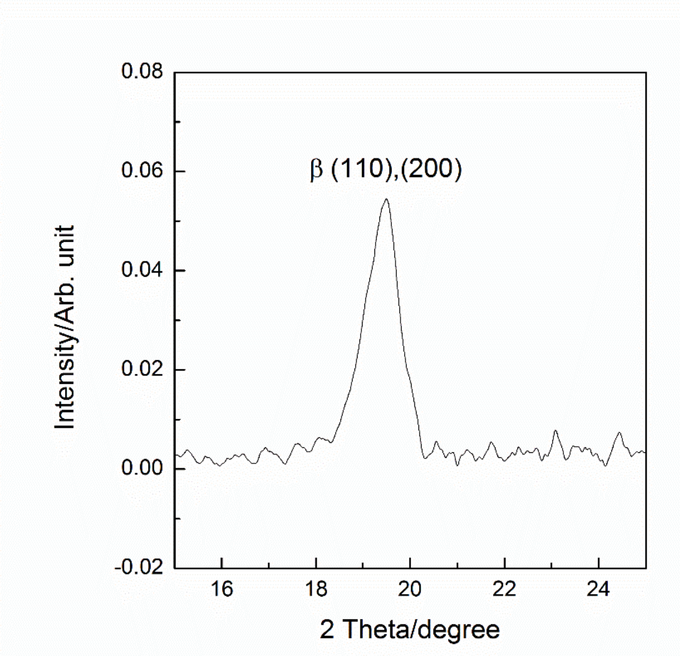

Figure S1. XRD spectrum of the P(VDF-TrFE) nanotube without residual film.

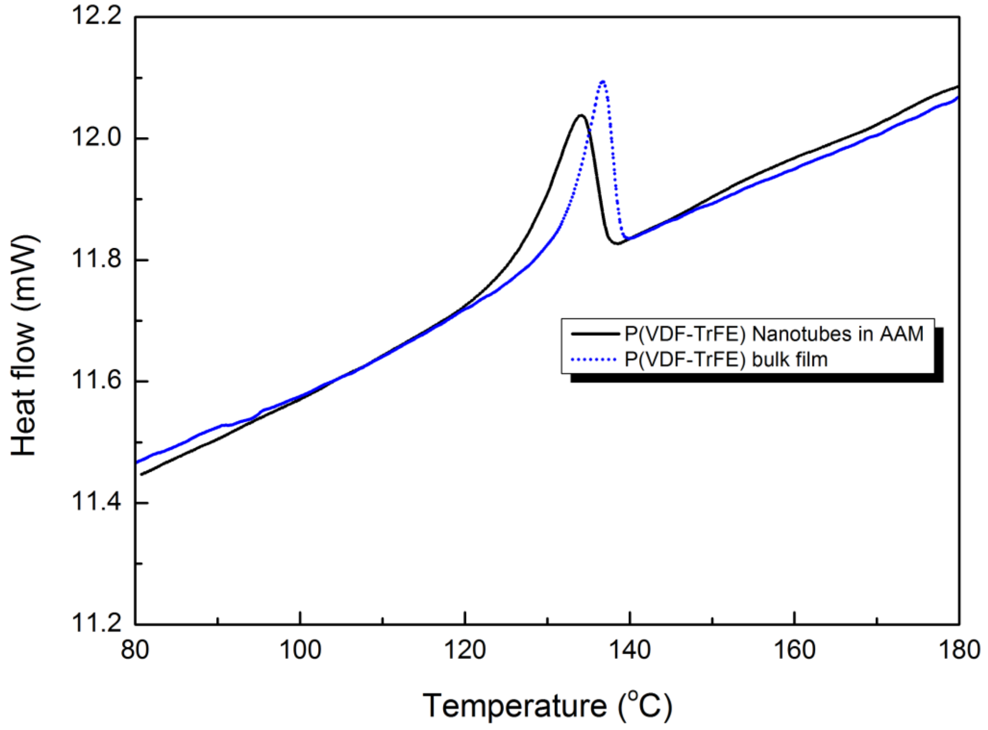

Figure S2. Cooling curve of DSC scan of P(VDF-TrFE) nanotubes in AAM templates and P(VDF-TrFE) bulk film (Cooling rate: 20 K min^{-1}).

Calculation of Total Polarization

The total polarization of nanotube array is modeled using an electric circuit as shown in Figure S3. The nanotube array is divided into three regions in parallel configuration. Region 1 consists of the remaining alumina from AAM template in series with P(VDF-TrFE) residual film. Region 2 consists of P(VDF-TrFE) from nanotube wall in series with residual film and Region 3 consists of the air in nanotube in series with residual film.

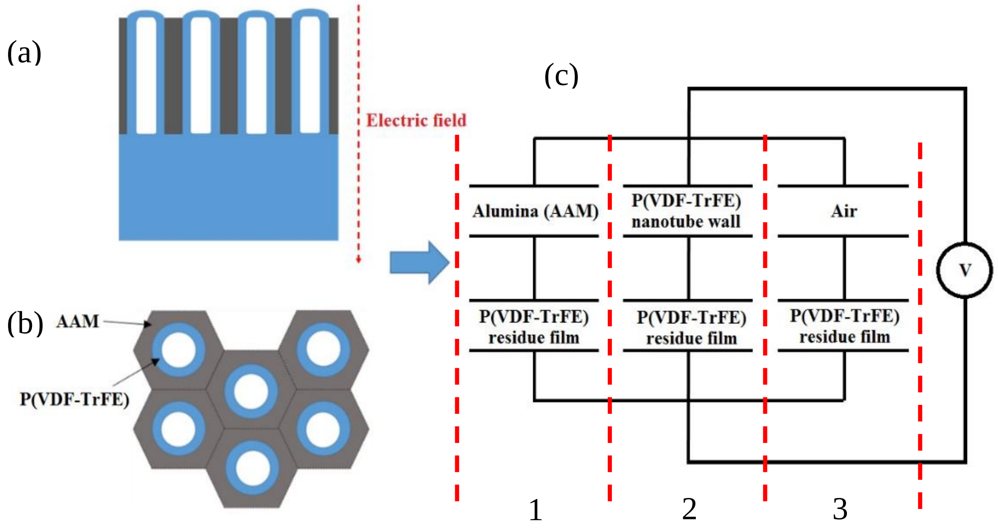

Figure S3 Cross section of nanotube array a) side view; b) top view; and c) the electrical model to calculate the total polarization.

Due to the presence of alumina and air in series connection with P(VDF-TrFE), the low charge carrier density of these materials causes insufficient compensation charge to move into close proximity of ferroelectric surfaces to completely eliminate the depolarization field. Hence, the incomplete shielding of depolarization field destabilize and reduce the ferroelectric polarization in the nanotube array. The magnitude of electric field in the ferroelectric due to incomplete shielding of depolarization field is

$$E = \frac{\sigma - P}{\varepsilon_0 \varepsilon_{ferro}} \qquad (1)$$

where P is the ferroelectric polarization, σ is the surface charge density on ferroelectric, and ε_{ferro} is the dielectric constants of the ferroelectric. By considering the ferroelectric and dielectric (air or alumina) in series connection, the voltage across the ferroelectric is

$$V = V_{ferro} + V_d = \frac{Q_{ferro} - PA}{C_{ferro}} + \frac{Q_d}{C_d} \qquad (2)$$

where V is the total voltage across the dielectric and ferroelectric, V_{ferro} and V_d are the voltage across the ferroelectric and dielectric respectively, Q_{ferro} and Q_d are the charge, C_{ferro} and C_d are the capacitance of ferroelectric and dielectric respectively. Q_{ferro} is equal to Q_d because of the conservation of charge. The amount of compensation charge available to shield the depolarization field in the presence of a series dielectric can be estimated by setting $V = 0$.

$$0 = \frac{Q_{ferro} - PA}{C_{ferro}} + \frac{Q_d}{C_d} \quad (3)$$

$$V_{ferro} = \frac{PA}{C_d + C_{ferro}} \quad (4)$$

For a simplified estimation of effective remnant polarization in the ferroelectric with insufficient compensation charge, the effective remnant polarization is assumed to have a linear relationship with the electric field inside the ferroelectric (V_{ferro}).[1]

$$P(V = 0) = P_r\left(1 - \frac{V_{ferro}}{V_c}\right) \quad (5)$$

where $P(V=0)$ is the effective remnant polarization and P_r is the remnant polarization with fully compensated depolarization field. By combining Equations (4) and (5), and considering the same thickness and area for both ferroelectric and dielectric in series, it can be obtained that

$$P(V = 0) = \frac{P_r}{1 + \frac{P_r}{(\varepsilon_d + \varepsilon_{ferro})\varepsilon_0 E_c}} \quad (6)$$

where E_c is the coercive field. The total effective remnant polarization for nanotube array can be estimated as the sum of effective remnant polarization in each regions. The effective remnant polarization of Regions 1 and 3 is calculated based on the depolarization field suppression due to the presence of alumina and air. The calculated effective remnant polarization of respective regions are shown in Table S1. Depolarization field for Region 2 is assumed to be fully compensated due to the direct contact of P(VDF-TrFE) with bottom and top Au electrodes. Previous quantitative study showed that the polarization in the P(VDF-TrFE) film with randomly oriented polymer chains is two-third of that in the film with all polymer chains aligned perpendicular to the applied electric field. Hence, the polarization in the P(VDF-TrFE) nanotubes is estimated to be approaching the theoretical limit of 11 μC cm^{-2} due to preferred orientation of polymer chains. The sum of the effective remnant polarization can be expressed as

$$P(V=0)_{total} = A_1P_1 + A_2P_2 + A_3P_3 \qquad (7)$$

where A_1, A_2 and A_3 are the ratio of the areas and P_1, P_2 and P_3 are the effective polarization of respective regions, as shown in Table S1. The calculated total effective remnant polarization is about 4.35 μC cm^{-2}.

Table S1 Ratio of the area and effective remnant polarization for respective regions.

Region	Area in one unit cell (nm^2)	Area ratio	P(V=0) (μC cm^{-2})
1	63,904	0.40	1.1
2	54,671	0.34	11.0
3	41,553	0.26	0.67

[1] C.T. Black, C. Farrel, & T. J. Licata, *Appl. Phys. Lett.* **1997**, 71, 2041.
